# Unveiling Drought Tolerant Cotton Genotypes: Insights from Morpho-Physiological and Biochemical Markers at Flowering

**DOI:** 10.3390/plants14040616

**Published:** 2025-02-18

**Authors:** Muhammad Yousaf Shani, Muhammad Yasin Ashraf, Muhammad Ramzan, Zafran Khan, Nimra Batool, Nimra Gul, William L. Bauerle

**Affiliations:** 1 Plant Breeding and Genetics Division, Nuclear Institute for Agriculture and Biology, Jhang Road, P.O. Box 128, Faisalabad 38000, Pakistan; mmyousafshani@gmail.com; 2 Pakistan Institute of Engineering and Applied Sciences (PIEAS), Nuclear Institute for Agriculture and Biology/College (NIAB-C), Islamabad 45650, Pakistan; muhammadramzan293@gmail.com; 3 Department of Plant Breeding and Genetics, University of Agriculture Faisalabad, Faisalabad 38000, Pakistan; zafrankhan.mandokhail@gmail.com (Z.K.); nimratahir122@gmail.com (N.G.); 4 Institute of Molecular Biology and Biotechnology (IMBB), The University of Lahore, Lahore 54000, Pakistan; nimrauol@gmail.com; 5 Department of Horticulture and Landscape Architecture, Colorado State University, Fort Collins, CO 80523, USA; bill.bauerle@colostate.edu

**Keywords:** biochemical indicators, cotton, drought stress, heatmap, MGIDI, PCA

## Abstract

Drought stress substantially restricts cotton growth, decreasing cotton production potential worldwide. This study evaluated cotton genotypes at the flowering stage to identify drought-resilient genotypes under moderate and severe drought conditions using physio-morphic and biochemical markers. Five genotypes were examined in a completely randomized design with three replicates across three treatments. Growth and biochemical traits were measured after 14 days of drought stress. The Multi-trait Genotype–Ideotype Distance Index (MGIDI) identified the most drought-tolerant genotypes. Severe drought had a pronounced negative effect on growth and biochemical traits, followed by moderate drought. Among the genotypes, FH-912 exhibited the strongest resilience, with significant increases in proline, peroxidase, catalase, and total chlorophyll. In contrast, chlorophyll a and transpiration rates were largely unaffected. Genotypes VH-351, VH-281, and GH-99 showed moderate drought tolerance, while FH-556 was highly sensitive to water stress. Statistical analyses, including ANOVA, PCA, and heatmaps, confirmed FH-912’s superior performance under drought stress. The drought-resilient genotype, FH-912, holds promise for breeding drought-tolerant cotton varieties to sustain cotton productivity in water-limited environments, especially in drought-prone regions.

## 1. Introduction

Abiotic and biotic stresses pose significant threats to global food security, disrupting the continuous food supply for developing populations [1]. Among abiotic stressors, drought stress is particularly detrimental, leading to a 50–70% decline in crop yield. Water scarcity affects approximately 55 million people annually [2], and projections indicate that drought stress could displace 700 million people globally by 2050 [3]. In Pakistan, water scarcity has severely impacted cotton cultivation in South Punjab, Sindh, and Baluchistan provinces due to low rainfall and erratic weather patterns [4]. The Indus River System Authority (IRSA) forecasts a 30–35% water shortfall for the upcoming Kharif season, threatening key crops, including cotton (*Gossypium hirsutum*) [5].

Cotton, often referred to as “white gold”, is globally considered a cash crop and a key natural fiber source for the textile industry [6]. The top five cotton-producing countries across the globe are Brazil, India, China, the United States, and Pakistan [7]. These nations contribute two-thirds of total global cotton production, estimated at 25 billion kg annually [8]. Cotton accounts for about 60% of Pakistan’s foreign earnings, making it the fourth-largest exporter of cotton yarn [9]. In the 2022–2023 season, production dropped to 4.91 billion kg, and the yield drastically fell to 390 kg/ha due to severe climatic changes [10]. As a glycophytic crop, cotton can tolerate moderate climatic stress. However, extreme drought stress poses a serious threat to cotton plants, disrupting osmotic balance and cellular metabolic processes [11] and leading to considerable morphological variation [12]. Over the past decade, a 14% decline in cotton yield has been observed, from 13.96 million bales to 11.93 million bales. Drought stress disrupts plant physiological processes by affecting water balance, gas exchange, and metabolism. It reduces stomatal conductance, leading to lower transpiration and photosynthetic efficiency, ultimately decreasing chlorophyll content, carbon assimilation, and energy production, negatively impacting growth and yield. To counteract these effects, plants activate defense mechanisms such as osmolyte accumulation (proline, glycine betaine, and soluble sugars) to maintain cellular turgor and protect membranes. Antioxidants are crucial in reducing the oxidative damage caused by reactive oxygen species (ROS) [13].

Cotton grown in a water-limited environment exhibits poor root development, reduced cottonseed productivity, and increased susceptibility to insect pests [14]. Moreover, the reproductive stage of cotton is more sensitive to water scarcity than the seedling stage, as water deficiency can impair pollen adhesion and fertilization of the developing ovary [15]. In Pakistan, the prevalence of heat stress and water scarcity has led to a 34% reduction in cottonseed yield [16]. Given limited water resources and increasing global water demand, developing drought-tolerant cotton varieties has become crucial and can be achieved through modern breeding strategies [17].

Cotton genetic variability possesses several pivotal characteristics that influence its response to water-limited environments [18]. Among physiological processes, stomatal conductance (gs), transpiration rate (E), and net photosynthetic activity (Pn) are key indicators of a cotton genotype’s adaptability to water deficit stress. Water scarcity significantly impairs the aforementioned physiological attributes due to drastic alterations in physio-morphological traits [19]. Extreme water scarcity also leads to a substantial decline in shoot length (SL), shoot fresh mass (SFM), root fresh mass (RFM), and plant dry mass [20]. Cotton drought tolerance is significantly associated with the ability to maintain membrane integrity and the relative water content (RWC) in leaves, which directly reflects a plant’s water status during shortages [21].

Cotton crops employ intricate biochemical mechanisms to mitigate the accumulation of ROS by producing enzymatic and non-enzymatic antioxidants [22]. Drought limits water availability, reducing stomatal conductance and photosynthetic efficiency, which leads to excess energy accumulation in chloroplasts and mitochondria. This excess energy promotes ROS generation, including superoxide radicals (O_2_^−^), hydrogen peroxide (H_2_O_2_), and hydroxyl radicals (OH^•^). Drought-induced metabolic disruptions in electron transport chains of chloroplasts, mitochondria, and peroxisomes further enhance ROS production [13].

To combat ROS toxicity, plants activate antioxidant defense mechanisms by producing enzymes such as superoxide dismutase (SOD), which converts superoxide radicals into hydrogen peroxide, catalase (CAT), which breaks down hydrogen peroxide into water and oxygen, and peroxidase (POD), which neutralizes ROS by utilizing electron donors. These antioxidant enzymes play a crucial role in protecting cellular structures, maintaining membrane integrity, and ensuring metabolic stability under drought stress [23].

Under severe water scarcity, resilient cotton accessions typically avoid drastic alterations in cell homeostasis, respiration, and photosynthesis, owing to less interference induced by ROS [24]. In contrast, susceptible cotton genotypes are unable to maintain an appropriate balance between antioxidants and ROS synthesis, leading to more disruption in cell membrane integrity, lipid peroxidation, and a reduction in lint yield [25].

The current research focuses on evaluating drought-tolerant cotton genotypes using modern physio-morphological and biochemical markers at the early flowering stage. While previous studies have primarily emphasized screening cotton germplasms at the seedling stage, the reproductive stage is more vulnerable to water scarcity. Therefore, this research aims to accurately identify drought-resilient cotton genotypes with improved boll retention and fiber productivity. Subsequently, the selected genotypes could be utilized in future breeding programs to develop drought-tolerant cotton varieties for water-limited areas.

## 2. Results

### 2.1. Growth Parameters

**Figure 1:**
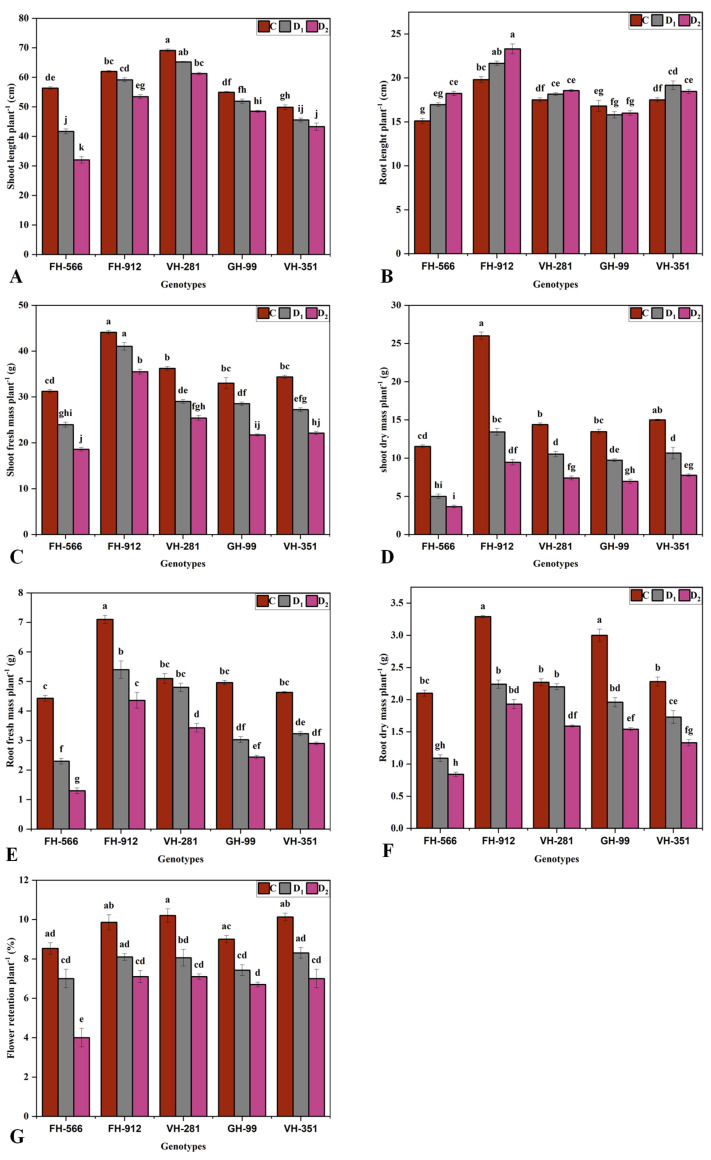
Effect of different water deficit levels on plant growth parameters and flower retention of five cotton genotypes. Plants were grown under control (100%), moderate (50%), and severe (30%) container water holding capacity conditions. Mean (**A**) shoot length, (**B**) root length, (**C**) shoot fresh mass, (**D**) root fresh mass, (**E**) shoot dry mass, (**F**) root dry mass, and (**G**) flower retention per plant. Bars represent the mean value (XX ± SE), where XX refers to the mean of three biological replicates. Different letters indicate statistical differences (*p* < 0.05) among treatment × genotype interactions.

Drought stress significantly influenced the growth parameters of all cotton genotypes in this study at the flowering stage under moderate (50%) and severe (30%) container water holding capacity conditions. Genotype × environment interactions were also significant for most parameters, except chlorophyll a and E (Table 1 and Table 2). Among the genotypes, VH-281 exhibited the greatest SL under both moderate (65.23 cm) and severe drought (61.2 cm) conditions, while FH-556 had the lowest SL (41.69 cm and 32.03 cm, respectively) (Figure 1A). For root length (RL), FH-912 excelled, maintaining the highest RL under moderate (21.6 cm) and severe drought stress (53.4 cm), whereas FH-556 consistently had the lowest values (16.9 cm and 18.2 cm, respectively) (Figure 1B). FH-912 also demonstrated a superior performance in terms of SFM under moderate (41.03 g) and severe drought stress (35.5 g). Conversely, FH-556 had the lowest SFM (23.9 and 18.6 g, respectively) (Figure 1C). Similarly, FH-912 had the highest RFM (5.4 and 4.3 g) under moderate and severe drought stress, while FH-556 had the lowest (2.3 and 1.3 g, respectively) (Figure 1E). Moreover, FH-912 had the highest shoot dry mass (SDM) (13.4 and 9.4 g) and root dry mass (RDM) (2.2 and 1.9 g) under moderate and severe drought conditions, whereas FH-556 displayed the lowest SDM (5 and 3.6 g) and RDM (1.09 and 0.8 g) (Figure 1D,F).

FH-912 exhibited the highest flower retention (FR) under moderate drought stress (8.1%), whereas FH-554 showed the lowest (7%). Under severe drought stress, both FH-912 and VH-281 maintained the highest FR (7.1%), whereas FH-554 had the lowest (4%) (Figure 1G). Overall, the GH-99, VH-281, and VH-351 genotypes exhibited moderate drought tolerance, maintaining relatively stable growth parameters under moderate and severe drought conditions (Figure 1).

### 2.2. Physio-Biochemical Parameters

**Figure 2:**
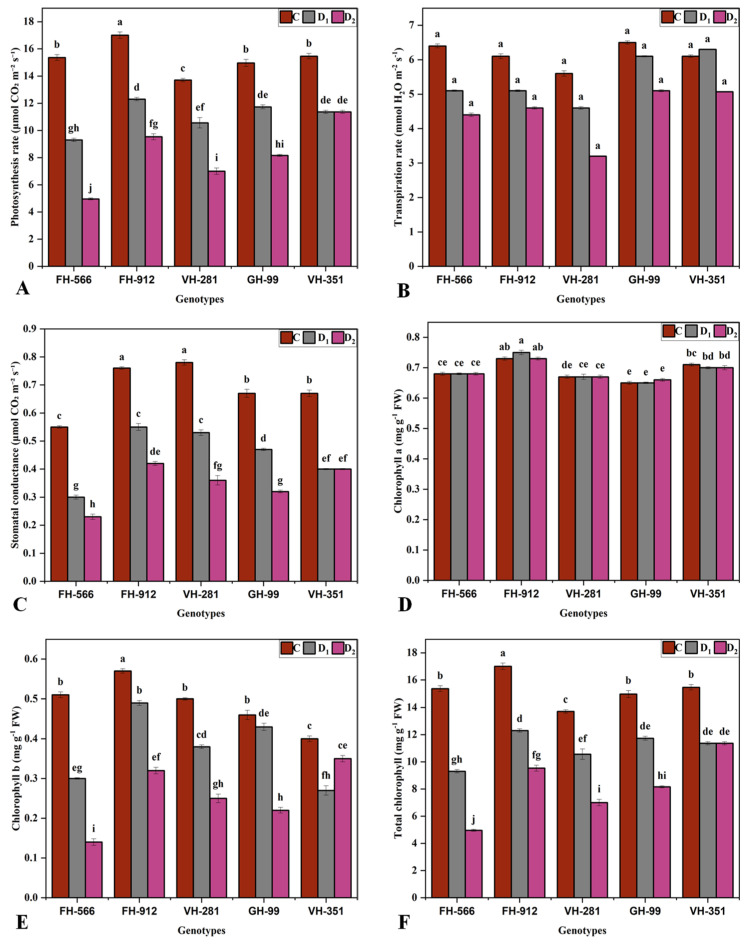
Effect of different water deficit levels on (**A**), photosynthesis rate (**B**), transpiration rate (**C**), stomatal conductance (**D**), chlorophyll a (**E**), chlorophyll b (**F**), and total chlorophyll per plant across five cotton genotypes grown under control (100%, C), moderate (50%, D1), and severe drought (30%, D2) relative to container water holding capacity conditions. Bars represent the mean value (XX ± SE), where XX refers to the mean of three biological replicates. Different letters indicate statistical differences (*p* < 0.05) among treatment × genotype interactions.

All measured parameters were significantly impacted (*p* ≤ 0.05) by moderate and severe drought stress (Figure 2). FH-912 maintained the highest Pn (12.3 and 9.5 μmol CO_2_ m^−2^ s^−1^) and gs (0.5 and 0.4 μmol CO_2_ m^−2^ s^−1^) under moderate and severe drought stress, respectively. In contrast, FH-556 had the lowest Pn (9.3 and 4.9 μmol CO_2_ m^−2^ s^−1^) and gs (0.3 and 0.2 μmol CO_2_ m^−2^ s^−1^) under drought stress conditions (Figure 2A,C). Chlorophyll a, remained unaffected by drought stress (*p* > 0.05), with no significant genotype × environment interaction (Figure 2D).

**Figure 3:**
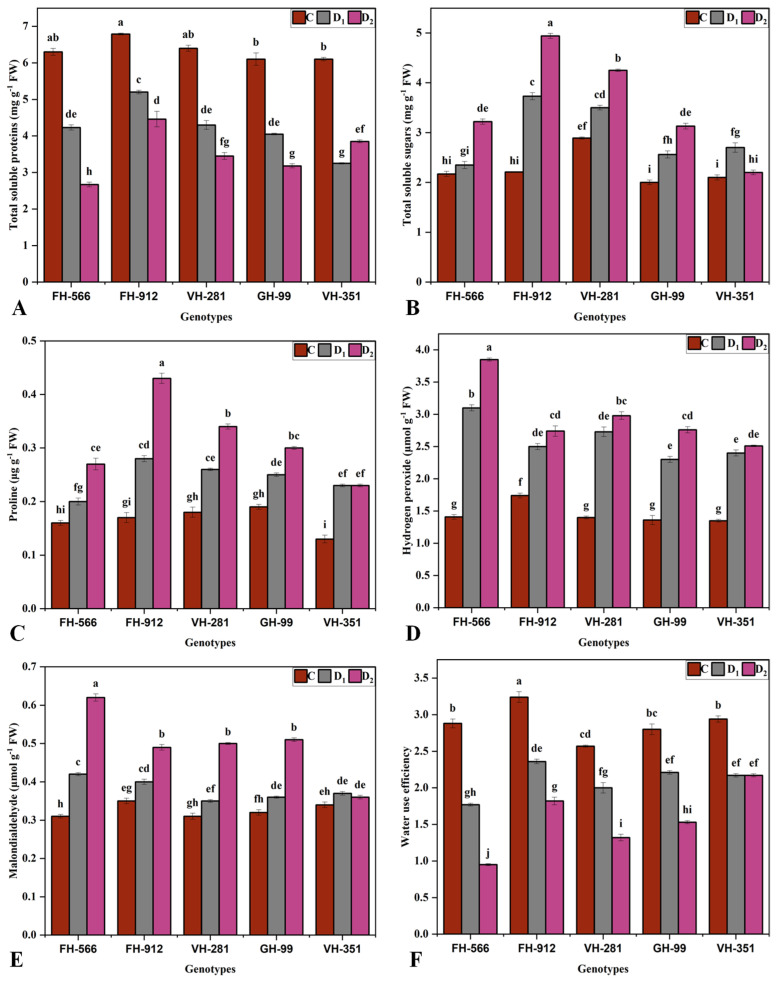
Effect of different water deficit levels on (**A**) total soluble proteins, (**B**) total soluble sugars, (**C**) proline, (**D**) hydrogen peroxidase, (**E**) malondialdehyde, and (**F**) water use efficiency per plant across five cotton genotypes grown under control (100%, C), moderate (50%, D1), and severe drought (30%, D2) relative to container water holding capacity conditions. Vertical bars represent the standard error of the mean. Bars represent the mean value (XX ± SE), where XX refers to the mean of three biological replicates. Different letters indicate statistical differences (*p* < 0.05) among treatment × genotype interactions.

FH-912 exhibited the highest chlorophyll b (Chl.b) (0.49 and 0.32 mg g^−1^ FW) and total chlorophyll (T.chl) (1.93 and 1.23 mg g^−1^ FW) concentrations under moderate and severe drought, respectively. In contrast, FH-556 had the lowest chlorophyll b (0.3 and 0.1 mg g^−1^) and T.chl (1.09 and 0.53 mg g^−1^) concentrations (Figure 2E,F). Similarly, FH-912 showed the highest total soluble protein (TSP) (5.2 and 4.4 mg g^−1^ FW) and total soluble sugar (TSS) (3.7 and 4.9 mg g^−1^) values, whereas FH-554 had the lowest TSP (4.2 and 2.6 mg g^−1^ FW) and TSS (2.3 and 3.9 mg g^−1^) values under moderate and severe water stress, respectively (Figure 3A,B). The proline content peaked in FH-912 (0.28 μg g^−1^ FW and 0.43 μg g^−1^ FW) under moderate and severe drought, while FH-556 had the lowest proline levels (0.2 and 0.27 μg g^−1^ FW) (Figure 3C). In addition, elevated levels of water use efficiency (WUE) were reported in FH-912 (2.36 and 1.82) under moderate and severe water deficits, whereas the lowest (1.77 and 0.95) was noted in FH-556 (Figure 3F).

Reactive oxygen species, namely H_2_O_2_ (Figure 3C) and MDA (Figure 3D), were significantly elevated under drought stress conditions. Under moderate drought, FH-556 had the highest H_2_O_2_ value (3.1 μmol g^−1^ FW), whereas GH-99 showed the lowest (2.3 μmol g^−1^ FW), closely followed by FH-912 (2.5 μmol g^−1^ FW) and VH-351 (2.4 μmol g^−1^ FW) (Figure 3D). Under severe drought, FH-556 had the highest H_2_O_2_ concentration (3.85 μmol g^−1^ FW), while FH-912 exhibited the lowest (2.74 μmol g^−1^ FW). Similarly, FH-556 had the highest MDA levels under both drought stress treatments, whereas the lowest concentrations were observed in GH-99 (0.36 μmol g^−1^ FW) under moderate drought and VH-351 (0.37 μmol g^−1^ FW) under severe drought stress.

**Figure 4:**
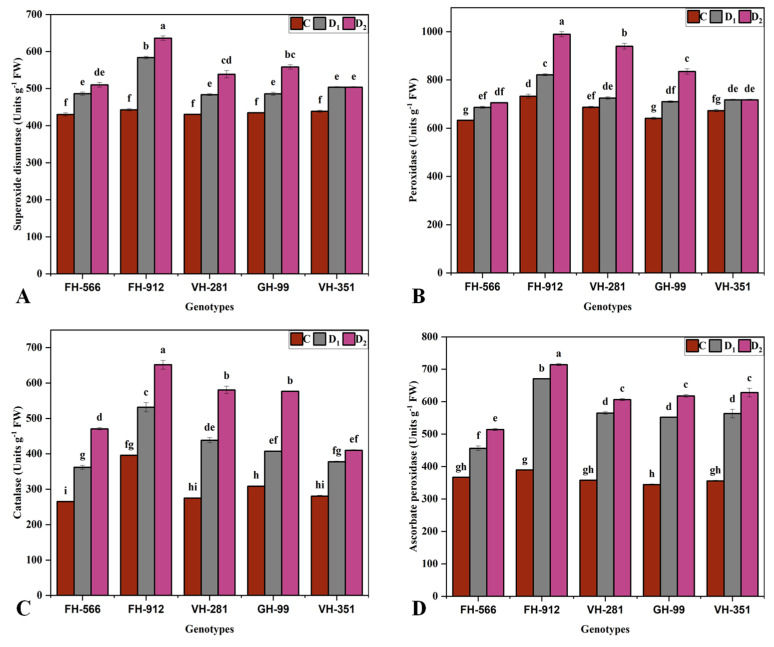
Effect of different water deficit levels on (**A**) superoxide dismutase, (**B**) peroxidase, (**C**) catalase, and (**D**) ascorbate peroxidase per plant across five cotton genotypes grown under control (100%), moderate (50%), and severe drought (30%) relative to container water holding capacity conditions. Vertical bars represent the standard error of the mean. Bars represent the mean value (XX ± SE), where XX refers to the mean of three biological replicates. Different letters indicate statistical differences (*p* < 0.05) among treatment × genotype interactions.

Drought stress treatments significantly influenced antioxidant enzyme activities across all cotton genotypes (*p* ≤ 0.05), with pronounced increases observed in the genotype FH-912. This genotype exhibited the highest SOD concentrations (583.6 and 636 units g^−1^ FW) and POD concentrations (821.6 and 989.3 units g^−1^ FW) under moderate and severe drought stress, respectively (Figure 4A,B). In contrast, GH-99 showed the lowest SOD activity (486 units g^−1^ FW), and FH-556 had the lowest POD activity (686.6 units g^−1^ FW) under moderate drought stress. FH-912 also showed peak CAT and APX activities, reaching 531.6 and 651.6 units g^−1^ FW for CAT and 651.6 and 714 units g^−1^ FW for APX under moderate and severe drought, respectively. Conversely, VH-351 exhibited the lowest CAT activity (362 units g^−1^ FW), while FH-556 showed the lowest APX activity (456.3 units g^−1^ FW) under moderate drought (Figure 4C,D). Under severe drought, VH-351 again had the lowest CAT activity (377.6 units g^−1^ FW), and FH-556 showed the lowest APX activity (514.3 units g^−1^ FW). The GH-99, VH-281, and VH-351 genotypes exhibited moderate antioxidant responses under drought stress. However, specific physio-biochemical traits, such as Chl. a and E, remained unaffected by the drought treatments (Figure 4).

### 2.3. Principal Component and Heatmap Analysis

**Figure 5:**
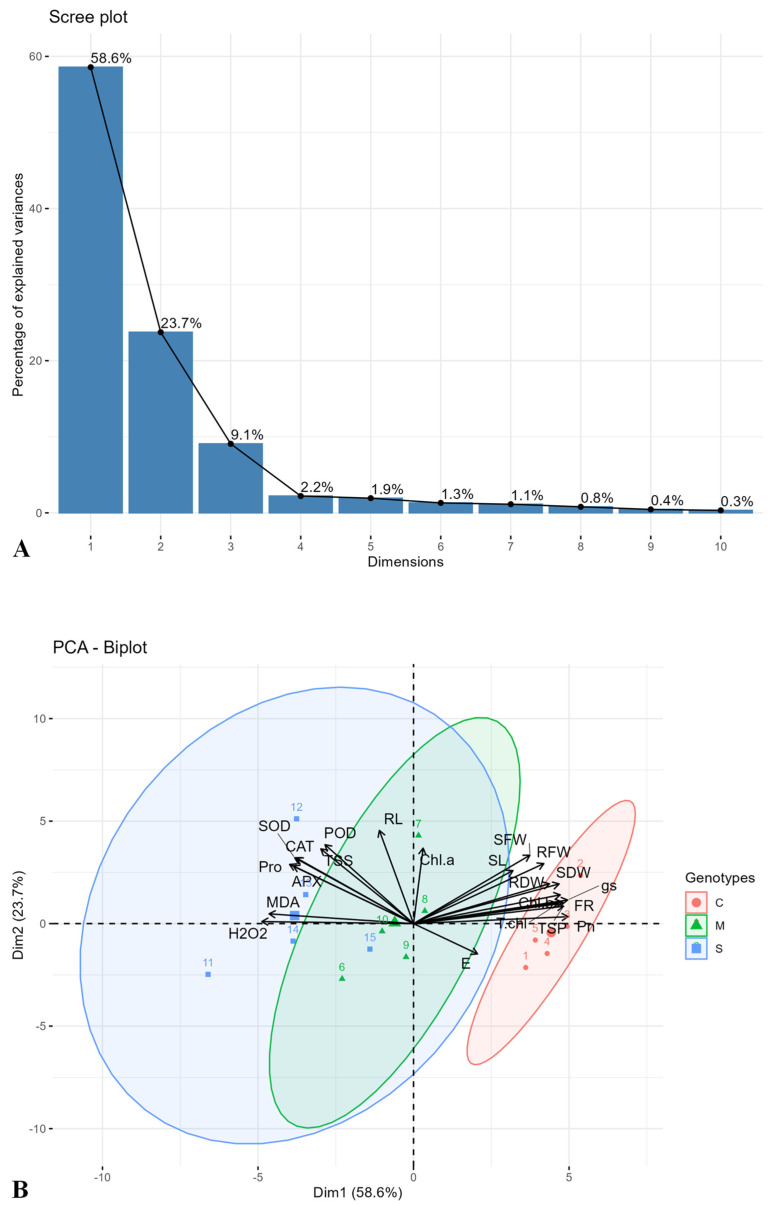
Scree plot analysis score values (**A**) show the percent variance contribution of each principal component towards cumulative variability, and biplot analysis (**B**) unveiled a graphical representation of physio-morphic and biochemical attributes across the five cotton genotypes under control, moderate, and severe drought stress conditions. The numeric values 1–5 show genotypes FH-556, FH-912, VH-281, GH-99, and GH-351 under control conditions, 6–10 under moderate drought stress, and 11–15 under severe drought stress, respectively. The vector length illustrates the relationship among variables and numeric values representing genotypes under control (●), moderate (▲), and severe drought stress (■) treatments.

The principal component analysis (PCA) focused on the first two principal components, PC-1 and PC-2, which collectively explained 82.3% of the total variance, with PC-1 accounting for 58.6% and PC-2 contributing 23.7% (Figure 5A). The PCA was conducted using factoextra and FactoMineR packages in R-software (version 4.3.1). The PCA biplot analysis revealed synergistic positive and negative interactions among the evaluated traits. PC-1 was primarily associated with several key traits, including SFM, RFM, SL, SDM, RDM, and FR. These traits exhibited a positive synergistic association with the genotype FH-912, as the arrows were directed in the same direction for the above-mentioned morphological traits. At the same time, they were negatively correlated with H_2_O_2_ and MDA levels, as their vectors were oriented in opposite directions, indicating stress-responsive divergence. In contrast, PC-2 highlighted enzymatic antioxidants (SOD, POD, CAT, and APX) that were positively correlated with one other. Elevated H_2_O_2_ levels were prominently observed in the genotype FH-556, showing its susceptibility to moderate and severe drought stress (Figure 5B).

### 2.4. Heatmap Analysis

**Figure 6:**
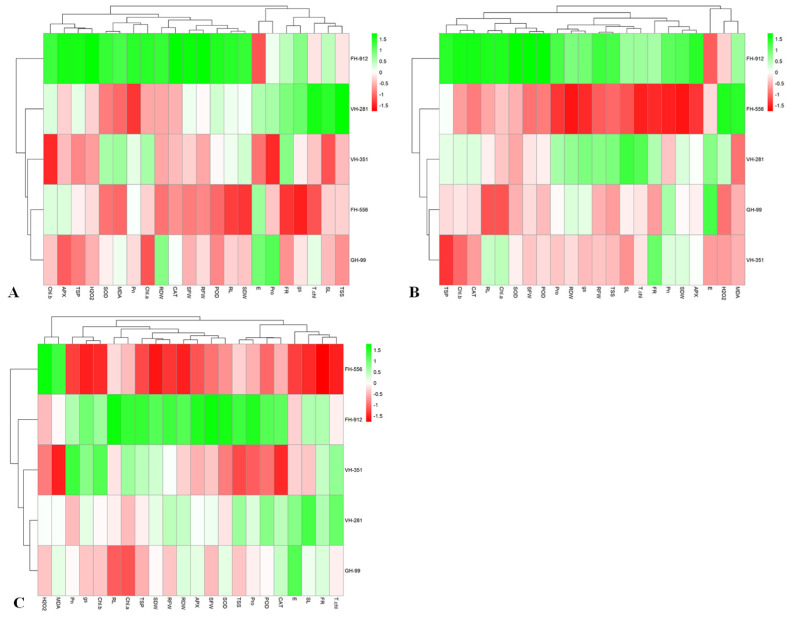
Heatmap analysis illustrating interactions among genotypes and traits in response to (**A**) control, (**B**) moderate, and (**C**) severe water stress conditions.

The heatmap revealed significant variations in the mean performance of cotton genotypes under control (Figure 6A), moderate (Figure 6B), and severe drought stress (Figure 6C). Under control conditions, the five genotypes were grouped into two distinct clusters, reflecting both positive and negative trait–genotype interactions. The FH-912 genotype exhibited a strong positive association with most physio-morphological and biochemical traits, except for E, highlighting its superior adaptability under non-stressed conditions compared to other genotypes (Figure 6A).

Under moderate drought stress, two distinct clusters emerged, highlighting a greater variability in genotype responses. FH-912 maintained a strong positive correlation with key growth and biochemical parameters, demonstrating its resilience. Conversely, FH-556 is negatively associated with most studied traits, indicating its susceptibility to moderate drought stress. The VH-281 and GH-99 genotypes exhibited moderate responses, sustaining growth and antioxidant activity, while VH-351 showed limited interaction with most traits except flower retention (Figure 6B).

Under severe drought conditions, FH-556 showed a pronounced negative interaction with critical traits, including FR, total chlorophyll, SL, shoot and root fresh mass, TSP, ascorbate peroxidase, and Pn. Simultaneously, it showed a strong positive connection with H_2_O_2_ and MDA, underscoring its sensitivity to drought stress. In contrast, FH-912 maintained positive interactions with most growth and biochemical parameters while exhibiting lower H_2_O_2_ and MDA levels, reaffirming its drought stress tolerance. The VH-351, GH-99, and VH-281 genotypes displayed moderate shifts in trait performance, reflecting their moderate drought tolerance (Figure 6C).

### 2.5. Multi-Trait Genotype–Idiotype Distance Index (MGIDI)

**Figure 7:**
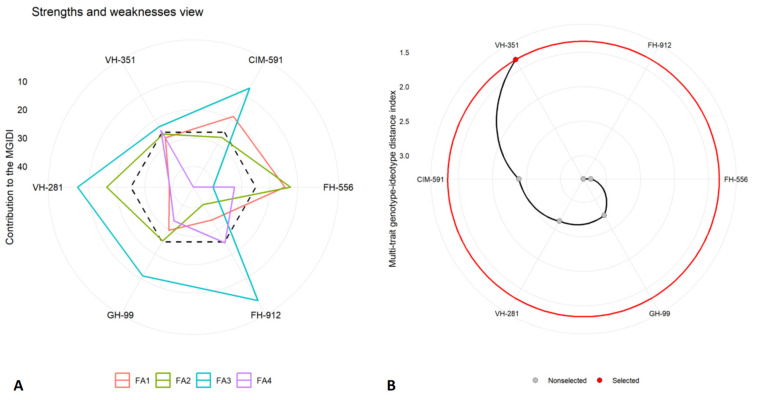
(**A**) Strengths and weaknesses under control conditions display the individual factor proportion in Multi-trait Genotype–Ideotype Distance Index (MGIDI) quantification. The closer the proportion of an illustrated factor is to the centroid area, the more the attributes within that factor align with the ideotype (ideal genotype). Conversely, a greater deviation of factor lines from the centroid indicates poorer performance for a specific set of traits in a particular genotype. The dotted line represents the theoretical value if all the factors contributed equally. (**B**) The ranking of genotypes is based on investigated traits, and the selected genotype is depicted with a black curve and red dot.

**Figure 8:**
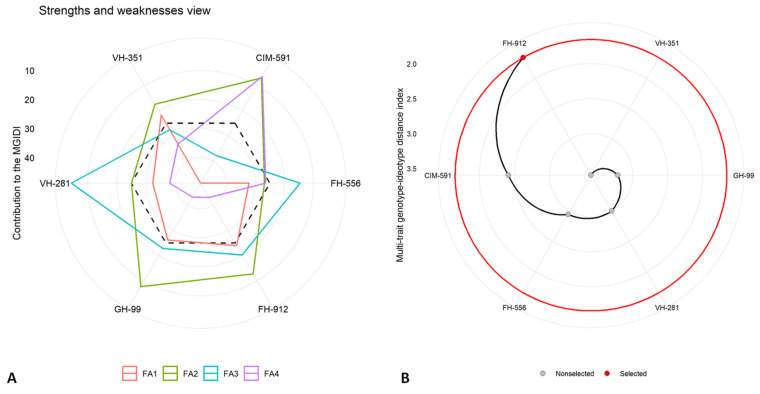
(**A**) Strengths and weaknesses under moderate drought stress display the individual factor proportion in Multi-trait Genotype–Ideotype Distance Index (MGIDI) quantification. The lower the proportion of an illustrated factor (nearer to the centroid area), the closer the attributes within the factors to the ideotype (ideal genotype), while a greater deviation in factor lines from the centroid indicates the poor performance for a specific set of traits for a particular genotype. The dotted line represents the theoretical value if all the factors contributed equally. (**B**) The ranking of genotypes is based on investigated traits, and the selected genotype is depicted with a black curve and red dot.

**Figure 9:**
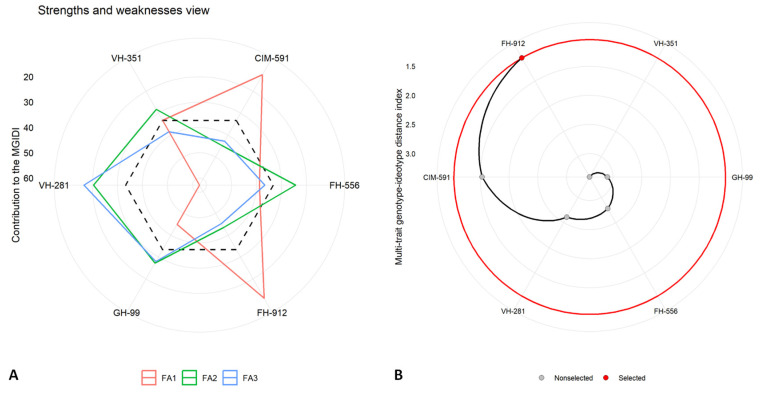
(**A**) Strengths and weaknesses under severe water scarcity display the individual factor proportion in Multi-trait Genotype–Ideotype Distance Index (MGIDI) quantification. The lower the proportion of an illustrated factor (nearer to the centroid area), the closer the attributes within the factors to the ideotype (ideal genotype), while more deviation in factor lines from the centroid indicates poor performance for a specific set of traits for a particular genotype. The dotted line represents the theoretical value if all the factors contributed equally. (**B**) The ranking of genotypes is based on investigated traits, and the selected genotype is depicted with a black curve and red dot.

The Multi-trait Genotype–Ideotype Distance Index (MGIDI) was employed to elucidate the top-performing cotton genotypes under varying water availability conditions. An MGIDI analysis, including genotype ranking and evaluations of strengths and weakness, was conducted separately for control (Figure 7), moderate (Figure 8), and severe drought stress (Figure 9) treatments at the flowering stage. This approach provided a comprehensive assessment of each genotype’s performance across the studied conditions, highlighting their respective strengths and limitations.

The results identified the FH-912 genotype as the most resilient under all conditions, consistently demonstrating a superior performance across moderate and severe drought stress scenarios. The MGIDI analysis highlighted specific traits in FH-912 that contributed to its resilience, emphasizing its adaptability under water-limited conditions. The factors (FAs) in the strength and weakness plot, such as FA_1_, FA_2_, FA_3_, and FA_4_, represent distinct sets of traits affecting genotype performance (Figure 7A, Figure 8A, and Figure 9A). Among the discrete latent factors, FA_1_ (red line) corresponds to growth-related attributes, FA_2_ (green line) encompasses physiological parameters, FA_3_ (blue line) represents antioxidant enzymes, and FA_4_ reflects the synthesis of reactive oxygen species. The factor lines emanate outward from the centroid region of the strength and weakness plot, indicating a poor performance of the relative traits for specific genotypes. In contrast, lines that are less scattered toward the peripheral region reveal a better performance for the corresponding set of studied traits. Additionally, the black dotted line represents the overall MGIDI score for each genotype. Higher values for a specific set of traits cause the FA to move farther from the center, indicating a greater deviation of a genotype from the ideal genotype. Under control, moderate, and severe water deficit scenarios, the factors were relatively close to the center or had lower MGID score values, highlighting FH-912 as the most stable genotype or one closest to the ideal genotype. Conversely, the VH-351, CIM 591, and GH-99 genotypes exhibited a moderate performance based on their deviation from the centroid toward the peripheral region. Despite this, the highest deviation across all studied traits was observed in the FH-556 genotype, which is considered the most susceptible cotton genotype.

Furthermore, the second panel (Figure 7B, Figure 8B, and Figure 9B) illustrates the ranking of genotypes based on their MGIDI score values or the total index. Under control conditions, the VH-351 genotype maintained optimal levels for all the traits studied. However, under moderate and severe drought stress conditions, the FH-912 genotype emerged as the most promising, remaining closest to the ideotype. This suggests that FH-912 could be utilized in future breeding programs to develop drought-tolerant cotton varieties (Supplementary Table S1). Additionally, high heritability (h^2^) values observed across all studied traits indicate a strong genetic basis, reinforcing the potential for significant selection gains under drought stress conditions (Supplementary Table S1). These findings further underscore the potential for these traits to be incorporated into breeding programs aimed at enhancing drought tolerance in cotton.

## 3. Discussion

Drought stress significantly affects crop growth and development by inducing substantial alterations in crop physio-biochemical processes, leading to marked yield reductions [26]. The results showed that drought stress severely impaired growth parameters, physiological responses, ROS concentrations, and antioxidant enzyme activities in cotton genotypes, demonstrating considerable variability in drought tolerance among genotypes. This variation highlights the genetic diversity for drought resilience within the studied cotton germplasm, aligning with findings in other crops in which genotypic differences influence stress tolerance [27].

The superior performance of FH-912 under both moderate and severe drought stress indicates its significant genetic potential for growth and physiological adaptability [28]. This genotype exhibited greater shoot and root lengths and superior shoot and root biomass accumulation under drought (Figure 1), traits critical for drought resistance due to their role in enhancing water and nutrient acquisition from the soil [29]. Notably, FH-912 maintained higher flower retention rates (Figure 1G), reflecting its capacity to sustain reproductive functions under limited water conditions. As flower retention directly influences yield potential [30], FH-912 emerges as a promising genotype for stable production under water stress conditions. The combination of these growth parameters with superior physiological traits in FH-912 highlights its robust stress tolerance mechanisms [31].

In cotton, physiological adaptations such as efficient photosynthesis (Figure 2A), transpiration rate (Figure 2B), and stomatal regulation (Figure 2C) are essential for optimizing WUE under drought conditions. The enhanced photosynthetic rate and gs observed in genotype FH-912 under stress conditions likely underpin its superior growth performance, as efficient photosynthesis under drought has been closely linked with sustained growth and biomass in water-limited environments [32]. Moreover, the elevated chlorophyll content in FH-912 (Figure 2F) indicates a maintained or adaptive photosynthetic pigment pool, which plays a crucial role in enhancing light absorption and energy conversion efficiency under stress, contributing to its overall drought resilience [33].

Drought stress significantly affected the physio-biochemical traits of all cotton genotypes, with FH-912 showing the highest Pn, gs, and WUE, indicative of an enhanced photosynthetic capacity under stress [34]. This capacity is likely supported by FH-912’s elevated levels of chlorophyll b (Figure 2E) and total chlorophyll (Figure 2F), essential pigments for effective light capture and energy conversion in photosynthesis [35]. These results suggest that FH-912’s photosynthetic machinery remains highly efficient and sustains better WUE under drought stress, a critical factor for maintaining growth and productivity when water availability is limited [36]. In contrast, genotype FH-556 showed the lowest values for Pn (Figure 2A), gs (Figure 2C), and chlorophyll content, reflecting a reduced photosynthetic efficiency and greater vulnerability to water stress. The intermediate performance observed in GH-99, VH-281, and VH-351 genotypes indicates moderate drought tolerance, as these genotypes maintain some photosynthetic and biochemical functionality despite drought constraints [32].

The variability observed in photosynthetic and biochemical attributes aligns with previous reports in the literature, in which genotypic differences in stress tolerance are commonly attributed to genetic architecture [37]. These genetic variations influence the efficiency of physiological processes such as stomatal regulation and pigment retention, which are critical for maintaining photosynthetic stability under stress [38]. A higher chlorophyll content, particularly chlorophyll b, enhances light absorption and energy transfer, optimizing photosynthetic activity in drought conditions [39]. Consequently, genotypes with higher chlorophyll concentrations, like FH-912, demonstrate greater resilience by sustaining metabolic and growth processes under water-limited conditions. The marked differences in photosynthetic traits and drought responses among the genotypes highlight the potential to utilize these physiological and biochemical markers in breeding programs [40]. Genotypes like FH-912, with enhanced photosynthetic and biochemical resilience, present promising candidates for developing drought-tolerant cultivars. These findings underscore the genetic basis for drought tolerance variation and highlight the importance of targeting photosynthetic efficiency, gs, and chlorophyll retention to improve cotton’s adaptability to water-stressed environments [41].

Drought-induced oxidative stress, marked by elevated levels of ROS such as H_2_O_2_ and MDA [42], was evident in all genotypes but was most pronounced in FH-556, which had the highest H_2_O_2_ and MDA levels (Figure 3D,E). High ROS concentrations disrupt cell membrane integrity, damage proteins and DNA, and hamper metabolic functions, which can lead to significant cellular damage, especially in drought-susceptible genotypes [42]. Conversely, FH-912 showed significantly lower ROS concentrations under drought conditions, suggesting an ability to prevent excessive oxidative buildup [43]. This reduced ROS accumulation in FH-912 appears to be mediated by an enhanced antioxidant defense system, as evidenced by higher activities of key antioxidant enzymes, including SOD (Figure 4A), POD (Figure 4B), CAT (Figure 4C), and APX (Figure 4D). These enzymes mitigate ROS damage by neutralizing superoxide radicals, converting H_2_O_2_ into water, and reducing peroxides [44]. The elevated activity of these enzymes in FH-912 reflects a robust antioxidative mechanism that aids cellular integrity and functionality during water stress. These findings align with research showing that drought-tolerant genotypes typically exhibit higher antioxidant enzyme activities, conferring resilience against oxidative damage (Figure 4) [45]. The results underscore the importance of antioxidant capacity (Figure 4) in managing ROS accumulation and minimizing cellular damage, establishing it as a key indicator of drought tolerance (Figure 3D,E). Thus, the elevated antioxidant activity observed in FH-912 confirms its classification as a drought-resilient genotype and demonstrates reduced ROS buildup and active ROS scavenging to sustain growth and physiological performance under drought conditions.

The PCA and heatmap analyses provided insights into genotypic responses to drought, distinguishing FH-912’s resilience from FH-556’s susceptibility (Figure 5 and Figure 6). The PCA biplot revealed that FH-912 clusters positively with key growth and biochemical traits under control, moderate, and severe drought conditions (Figure 5). These positive associations, especially with antioxidants and growth-related traits such as shoot and root biomass (Figure 1), FR (Figure 1G), Pn (Figure 2A), and antioxidant enzyme activity (Figure 4), highlight that FH-912 improved levels of physiological resilience and adaptability. In contrast, FH-556 was consistently grouped with stress susceptibility markers, including elevated H_2_O_2_ (Figure 3D) and MDA levels (Figure 3E), which are responsible for oxidative stress and cellular damage. The heatmap analysis corroborated these findings, showing that FH-912 maintained synergistic interactions with essential traits across imposed treatments, whereas FH-556 showed antagonistic correlations with key attributes, including SL, RFM, T.chl, and FR under drought (Figure 6). The observed clustering patterns align well with established findings that drought-tolerant genotypes typically exhibit positive interactions with antioxidants and growth parameters under stress conditions, enabling effective ROS management and the mitigation of oxidative damage [46]. Studies affirm that such associations enable plants to maintain cellular integrity, preserve photosynthetic efficiency, and sustain biomass production in drought scenarios [47]. FH-912’s alignment with these traits reinforces its classification as a drought-resilient genotype, providing a model for identifying and developing stress-tolerant cotton cultivars.

The MGIDI analysis also identified FH-912 as the most drought-tolerant genotype under moderate and severe drought stress (Figure 8 and Figure 9). In contrast, the genotype VH-351 exhibited a superior performance under control conditions (Figure 7). By consolidating multiple traits into a single performance score, the MGIDI highlighted FH-912’s drought tolerance, which was associated with a high level of heritability for growth, physiological, and biochemical traits (Supplementary Table S1). The strong heritability and selection differential of these traits suggest that FH-912’s favorable characteristics are genetically stable and reliably expressed under stress, making it a promising candidate for breeding drought-tolerant cotton varieties [48]. The GH-99, VH-281, and VH-351 genotypes demonstrated moderate drought tolerance, reflected in their intermediate MGIDI scores (Figure 8 and Figure 9). Although these genotypes exhibited some drought tolerance, their performance was inferior to FH-912 in key traits such as shoot and root biomass (Figure 1), antioxidant enzyme activity (Figure 4), and photosynthetic efficiency (Figure 2A). Nevertheless, these genotypes may serve as valuable genetic resources for breeding programs focused on incremental improvements in drought resilience [49]. In contrast, FH-556 consistently showed a strong negative association with critical growth (Figure 1) and biochemical parameters (Figure 3), underscoring its susceptibility to drought stress [50]. Its poor performance across multiple traits, reflected in higher MGIDI scores, highlights its limited adaptive capacity and reduced stress response under water deficit conditions [51]. The MGIDI approach effectively distinguished high-performing genotypes like FH-912 from lower-resilience genotypes such as FH-556, demonstrating its utility in genotype selection for drought tolerance breeding programs [52]. The findings align with previous studies emphasizing the role of genetic makeup in shaping drought responses across genotypes [53]. Numerous studies support the observation that drought-tolerant genotypes exhibit high levels of heritability and stability for traits, such as robust antioxidant enzyme activity, sustained photosynthesis, and consistent growth under stress [54]. These traits, often inherited across generations, are critical for plant survival in arid environments and are the focus of breeding efforts to enhance drought resilience [55].

## 4. Conclusions

The current investigation identified the cotton genotype FH-912 as a highly drought-tolerant cotton genotype, demonstrating exceptional resilience across morpho-physiological and biochemical traits. The robust growth performance of the FH-912 genotype, its efficient antioxidant responses, and its stable biochemical attributes under drought stress highlight its adaptability to water-limited conditions. This genotype maintained a higher photosynthetic efficiency and chlorophyll content and exhibited effective ROS scavenging, which minimized oxidative damage and supported sustained productivity under moderate and severe drought stress. These findings emphasize the critical role of selecting genotypes with a combined resilience in growth parameters, physiological stability, and antioxidant capacity to optimize cotton productivity in arid and semi-arid regions where water scarcity threatens agricultural yields. The adaptability of the FH-912 genotype implies its suitability for cultivation in these challenging environments, potentially offering a reliable cotton cultivar for farmers in drought-prone areas. Additionally, this research emphasizes the value of comprehensive selection criteria, including growth, biochemical, and stress response traits, in breeding programs aimed at developing drought-resilient cotton varieties. Such integrated approaches will be essential in promoting sustainable cotton production and mitigating the adverse impacts of climate change on crop yields. In conclusion, the present work was able to identify contrasting genotypes for drought stress, and these results could be useful for obtaining specific segregant populations for the identification of quantitative trait loci (QTLs) related to drought resilience in cotton.

## 5. Materials and Methods

The present research was conducted at the University of Agriculture, Faisalabad, Pakistan, using a completely randomized design (CRD) with three replicates across three treatments: control, moderate, and severe drought stress. Healthy seeds of five cotton genotypes (FH-912, VH-281, VH-351, GH-99, and FH-566) were obtained from the University of Agriculture Faisalabad, Pakistan, and Ayub Agriculture Research Institute, Faisalabad, Pakistan. Soil analyses were conducted before planting to determine container capacity (CC) and physio-chemical characteristics, including pH, electrical conductivity (EC), texture, and organic matter content (Table 3).

### 5.1. Selection of Drought-Tolerant Cotton Genotypes

Drought-tolerant cotton genotypes were identified based on key morpho-physio-biochemical traits. Morphologically, deep-rooted systems, better root biomass, moderate plant height, and reduced leaf area contributed to improved drought adaptation. Physiological traits such as higher RWC, regulated gs, efficient WUE, and stable chlorophyll content indicate sustained physiological performance under drought stress. Biochemical indicators include increased proline accumulation for osmotic adjustment, enhanced antioxidant enzyme activity (SOD, CAT, and POD) to mitigate oxidative stress, lower malondialdehyde (MDA) levels ensuring membrane stability, and higher soluble sugar concentrations for metabolic adjustments. Collectively, these traits were used as reliable selection criteria for identifying drought-tolerant cotton genotypes. Moreover, validation of crucial traits was executed using key statistical analyses such as MGIDI, PCA, and heatmap analyses.

### 5.2. Experimental Setup

The experiment occurred from 10 March 2023 to 15 May 2023 in a glass house under control conditions, with temperatures ranging from 35 ± 2 °C during the day and 28 ± 2 °C at night. Humidity was maintained at 70% using a humidifier and the help of cooling pads. To ensure optimal container capacity for seed sowing, 50 × 20 cm plastic containers were filled with a mixture of 6 kg clay and 1 kg sand (totaling 10 kg of substrate), irrigated with tap water, and left to drain to container capacity. After soaking the seeds for 12 h, four to five cotton seeds were sown in each pot at a depth of 2.5 cm. To enhance seedlings’ growth, five urea grains were added to each pot before sowing. Two weeks after germination, the first thinning was performed to retain only three seedlings per pot, followed by a second thinning to a single seedling five days later. The germinating seedlings were continuously irrigated with tap water until the initiation of the flowering stage. At the flowering stage, three moisture levels were maintained: 100%, 50%, and 30% CC applied 50 days after sowing during the early flowering stage. Growth and biochemical traits were measured after 14 days of drought stress.

### 5.3. Morphological Parameters

At the end of stress treatments, morphological traits were determined, including SL, RL, SFM, SDM, RFM, and RDM. Root and shoot lengths were measured in centimeters. An electronic weighing balance (model FA2104B) was utilized to determine the fresh weight of both roots and shoots. Subsequently, root and shoot sections were dried for four days at 72 °C in a reinforced heating oven (YPO-072) until constant weight was reached and then measured. FR in cotton is measured by assessing the number of flowers retained on the plant over a specific period. A fixed number of healthy and uniform cotton plants were selected per pot and the plants were tagged for each replication. At the initiation of flowering, freshly opened flowers were tagged using color-coded plastic tags or strings. Each tagged flower was marked with the date of anthesis to track its retention or abscission over time. Observations were recorded at regular intervals, typically every 7–10 days, until boll formation. The total number of tagged flowers at the time of tagging was counted, and after a defined period (20 days post-anthesis), retained flowers were counted and FR (%) was calculated using the following formula:

FR (%) = [(total number of tagged flowers − Flower after a defined period)/Total number of tagged flowers] × 100

### 5.4. Physiological Parameters

The physiological parameters of cotton genotypes were assessed under control (100% CC), moderate (50% CC), and severe drought stress (30% CC) conditions using leaf level infrared gas analysis (Ci-340, CID, Inc., Camas, WA, USA) to determine E, Pn, and gs. On sunny days, data were collected on the youngest fully expanded leaf of each replication per genotype after measurements stabilized between 10:00 am and 12:30 pm. Leaves were measured under the following conditions: ambient CO_2_ conditions, molar air flow per unit leaf area of 403.3 mmol m^2^ s^−1^, atmospheric pressure of 99.9 kPa, and water vapor pressure ranging from 6.0 to 8.9 mbar inside the chamber. The photosynthetically active radiation (PAR) at the leaf surface ranged from 1160 to 1350 μmol m^2^ s^−1^ and leaf temperature from 35 to 42 °C, whereas ambient temperature ranged from 35 to 40 °C.

### 5.5. Biochemical Analysis

The biochemical parameters were assessed using three plants for each treatment. SOD activity was determined by measuring its ability to inhibit the photochemical reduction of nitroblue tetrazolium (NBT) in the presence of superoxide radicals. The reaction mixture contained plant extract, riboflavin, NBT, phosphate buffer, and methionine, and the absorbance was measured at 560 nm [56]. POD content was determined using a guaiacol-based assay, in which the enzyme catalyzes the guaiacol oxidation in the presence of H_2_O_2_**.** The increase in absorbance due to guaiacol oxidation was measured at 470 nm. CAT activity was assessed by measuring the rate of H_2_O_2_ decomposition. The reaction mixture contained plant extract and H_2_O_2_ in phosphate buffer, and the decrease in absorbance was measured at 240 nm [57]. APX activity was quantified by measuring the decrease in absorbance at 290 nm as ascorbate is oxidized by H_2_O_2_ in the presence of plant extract [58]. MDA content, an indicator of lipid peroxidation, was measured by the thiobarbituric acid reactive substances (TBARS) assay. A mixture of plant extracts with TBA reagent was prepared and heated, and the absorbance of the resulting MDA-TBA complex was measured at 532 nm [59]. H_2_O_2_ levels were delineated by reacting plant extract with potassium iodide (KI) in an acidic medium. The color intensity was measured at 390 nm [60]. TSP concentration was determined using the Bradford assay, in which plant extract was mixed with Coomassie brilliant blue dye, and the absorbance was measured at 595 nm [61]. TSS was determined using the anthrone method. The plant extract was reacted with anthrone reagent, and the absorbance of the green-colored complex was measured at 620 nm [62]. Proline content was measured using the acidic ninhydrin method, with the colored complex measured at 520 nm [63]. Photosynthetic pigments (Chl a, Chl b, and T.chl) were extracted in 80% acetone, and their concentrations were calculated by measuring absorbance at 663 nm, 645 nm, and 470 nm, respectively, using specific equations for each pigment [64].

### 5.6. Statistical Analyses

A two-way analysis of variance (ANOVA) followed by Tukey’s HSD test was performed using XLSTAT (version 2020, Paris, France) to determine significant differences among means across treatments and genotypes. A scree plot was generated to visualize the contribution of each principal component (Figure 6A), and biplot analysis was performed for the first two principal components (Figure 6B). PCA was conducted on the physio-morphological and biochemical attributes to ascertain genotypes exhibiting strong positive associations with antioxidants and growth traits under drought conditions. Additionally, to assess variability among the five cotton genotypes, a PCA was conducted using the factoextra and FactoMineR packages in R (version 4.3.1) [65]. Heatmap analysis was performed using R software (version 4.4.2) and the Pheatmap package to evaluate variable associations under normal, moderate, and severe drought stress conditions [66]. Furthermore, heatmap analysis was conducted for each treatment using the Pheatmap package in R, pinpointing the interactions among observed traits and genotypes. The heatmap also facilitated the grouping of genotypes based on their responses to the applied treatments. The drought-tolerant genotype was selected by calculating the MGIDI using the metan package in R [67], with analyses conducted individually for genotypes grown under control, moderate, and severe drought stress.

## Figures and Tables

**Table 1:** Mean sum of squares for the investigated morpho-physiological and biochemical traits under control, moderate, and severe water stress conditions.

Source	DF	SL	RL	SFM	SDM	RFM	RDM	FR	Pn	E	Gs	Chl.a	Chl.b
**G**	4	710.5 ***	39.2 ***	321.2 ***	51.5 ***	11.3 ***	1.6 ***	6.4 ***	0.01 ***	0.01 ^ns^	0.06 ***	0.009 ***	0.2 ***
**T**	2	434.3 ***	9.1 ***	463.5 ***	200.5 ***	21.7 ***	5.0 ***	36.8 ***	0.08 ***	0.008 ^ns^	0.4 ***	0.0001 ^ns^	1.8 ***
**G × T**	8	45.9 ***	2.7 ***	3.7 *	1.3 *	0.5 **	0.1 ***	0.8 ^ns^	0.003 ***	0.006 ^ns^	0.004 ***	0.0001 ^ns^	0.05 ***
**Error**	28	2.01	0.4	1.41	0.53	0.08	0.01	0.4	0.0001	0.008	0.0003	0.00013	0.002
**Total**	44												

Significant differences among the drought stress treatments: *p* < 0.001 ***, *p* < 0.01 **, *p* < 0.05 *, and ns (non-significant); G, genotype; T, treatment; G × T, genotype and treatment interaction; DF, degree of freedom; SL, shoot length; RL, root length; SFM, shoot fresh mass; SDM, shoot dry mass; RFM, root fresh mass; RDM, root dry mass; FR, flower retention; Pn, net photosynthesis rate; E, transpiration rate; gs, stomatal conductance; Chl.a, chlorophyll a; and Chl.b, chlorophyll b.

**Table 2:** Mean sum of squares for the investigated morpho-physiological and biochemical attributes under normal, moderate, and severe water scarcity.

Source	DF	T.chl	WUE	TSP	TSS	Pro	SOD	POD	CAT	APX	H_2_O_2_	MDA
**G**	4	0.2 ***	6.7 ***	1.9 ***	3.3 ***	0.01 ***	9284 ***	42,725 ***	45,154 ***	24,863 ***	0.6 ***	0.009 ***
**T**	2	1.81 ***	0.65 ***	32.3 ***	6.1 ***	0.08 ***	50,093 ***	103,839 ***	192,437 ***	247,140 ***	9.4 ***	0.1 ***
**G × T**	8	0.05 ***	0.15 ***	0.5 ***	0.7 ***	0.003 ***	2387 ***	9524 ***	5806 ***	4925 ***	0.2 ***	0.008 ***
**Error**	28	0.002	0.008	0.02	0.01	0.00019	93	183	164	140	0.01	0.0001
**Total**	44											

Significant differences among the drought stress treatments: *p* < 0.001 ***; G, genotype; T, treatment; G × T, genotype and treatment interaction; DF, degree of freedom; T.chl, total chlorophyll; WUE, water use efficiency; TSP, total soluble proteins; TSS, total soluble sugars; Pro, proline; SOD, superoxide dismutase; POD, peroxidase; CAT, catalase activity; APX, ascorbate peroxidase; H_2_O_2_, hydrogen peroxide; and MDA, malondialdehyde.

**Table 3:** The physio-biochemical properties of the soil used to fill the pots in this experiment.

Soil Attributes	Values
Soil texture	Clay loam
Saturation percentage (%)	40.2–44.7
EC (dS m^−1^)	1.72–1.93
pH	7.53–7.81
Organic matter (%)	0.42–0.73
Mg + Ca (meq L^−1^)	2.63–4.84
CO_3_ (meq L^−1^)	None
HCO_3_ (meq L^−1^)	2.52–4.83
NO_3_-N (mg kg^−1^)	3.54–4.52
Total nitrogen (g kg^−1^)	0.43–0.52
Available K (mg kg^−1^)	75.5–80.5
Available P (mg kg^−1^)	1.52–2.83

## Data Availability

Data will be made available upon reasonable request to the corresponding author.

## Supplementary Materials

The following supporting information can be downloaded at: https://www.mdpi.com/article/10.3390/plants14040616/s1, Table S1: Association of factors to traits, original value (X_o_), selected value (Xs), selection Differential (SD), selection Differential percentage (SD%), heritability (h^2^), selection gains (SG), selection gain percentage (SG%), sense and goal under control, moderate and severe drought stress.

## References

[1] Roberts D.P., Mattoo A.K. (2018). Sustainable agriculture—Enhancing environmental benefits, food nutritional quality and building crop resilience to abiotic and biotic stresses. Agriculture.

[2] Khilchevskyi V., Karamushka V. (2021). Global water resources: Distribution and demand. Clean Water and Sanitation.

[3] Ahluwalia O., Singh P.C., Bhatia R. (2021). A review on drought stress in plants: Implications, mitigation and the role of plant growth promoting rhizobacteria. Resour. Environ. Sustain..

[4] Ashraf S., Ch K.M., Ashraf I., Akbar N. (2024). A phenomenological inquiry into farmers’ experiences growing cotton in Punjab, Pakistan. Sci. Rep..

[5] Ahmad S., Ahmad G. (2023). Water supply and demand: National and regional trends. Water Policy in Pakistan: Issues and Options.

[6] Aslam S., Khan S.H., Ahmed A., Dandekar A.M. (2020). The tale of cotton plant: From wild type to domestication, leading to its improvement by genetic transformation. Am. J. Mol. Biol..

[7] Shuli F., Jarwar A.H., Wang X., Wang L., Ma Q. (2018). Overview of the cotton in Pakistan and its future prospects. Pak. J. Agric. Res..

[8] Muhammad A., Smith S.A., Yu T.H.E. (2021). COVID-19 and cotton import demand in China. Agribusiness.

[9] Ali M.A., Farooq J., Batool A., Zahoor A., Azeem F., Mahmood A., Jabran K. (2019). Cotton production in Pakistan. Cotton production.

[10] Farooq M.A., Chattha W.S., Shafique M.S., Karamat U., Tabusam J., Zulfiqar S., Shakeel A. (2023). Transgenerational impact of climatic changes on cotton production. Front. Plant Sci..

[11] Zahid Z., Khan M.K.R., Hameed A., Akhtar M., Ditta A., Hassan H.M., Farid G. (2021). Dissection of drought tolerance in upland cotton through morpho-physiological and biochemical traits at seedling stage. Front. Plant Sci..

[12] Viot C.R., Wendel J.F. (2023). Evolution of the cotton genus, Gossypium, and its domestication in the Americas. Crit. Rev. Plant Sci..

[13] Rashid M.E., Islam M.A., Kanon T.A., Khan M.R., Uddin M.B., Haque R.U., Tonmoy M.W., Hasan M.R., Haque M.M. (2024). Valorization of dyed brush fiber waste through production of upcycled mélange yarn: A sustainable approach. J. Clean. Prod..

[14] Conaty W.C., Broughton K.J., Egan L.M., Li X., Li Z., Liu S., Llewellyn D.J., MacMillan C.P., Moncuquet P., Rolland V. (2022). Cotton breeding in Australia: Meeting the challenges of the 21st century. Front. Plant Sci..

[15] Arshad A., Raza M.A., Zhang Y., Zhang L., Wang X., Ahmed M., Habib-ur-Rehman M. (2021). Impact of climate warming on cotton growth and yields in China and Pakistan: A regional perspective. Agriculture.

[16] Mubarik M.S., Ma C., Majeed S., Du X., Azhar M.T. (2020). Revamping of cotton breeding programs for efficient use of genetic resources under changing climate. Agronomy.

[17] Sinha R., Fritschi F.B., Zandalinas S.I., Mittler R. (2021). The impact of stress combination on reproductive processes in crops. Plant Sci..

[18] Mahmood T., Khalid S., Abdullah M., Ahmed Z., Shah M.K.N., Ghafoor A., Du X. (2019). Insights into drought stress signaling in plants and the molecular genetic basis of cotton drought tolerance. Cells.

[19] Khadka K., Earl H.J., Raizada M.N., Navabi A. (2020). A physio-morphological trait-based approach for breeding drought tolerant wheat. Front. Plant Sci..

[20] Shelke D.B., Pandey M., Nikalje G.C., Zaware B.N., Suprasanna P., Nikam T.D. (2017). Salt responsive physiological, photosynthetic and biochemical attributes at early seedling stage for screening soybean genotypes. Plant Physiol. Biochem..

[21] Yang X., Lu M., Wang Y., Wang Y., Liu Z., Chen S. (2021). Response mechanism of plants to drought stress. Horticulturae.

[22] Sachdev S., Ansari S., Ansari M., Fujita M., Hasanuzzaman M. (2021). Abiotic stress and reactive oxygen species: Generation, signaling, and defense mechanisms. Antioxidants.

[23] Sarker U., Oba S. (2018). Catalase, superoxide dismutase and ascorbate-glutathione cycle enzymes confer drought tolerance of Amaranthus tricolor. Sci. Rep..

[24] Bhattacharya A., Bhattacharya A. (2021). Effect of soil water deficit on growth and development of plants: A review. Soil Water Deficit and Physiological Issues in Plants.

[25] Hasanuzzaman M., Raihan M.R.H., Masud A.A.C., Rahman K., Nowroz F., Rahman M., Nahar K., Fujita M. (2021). Regulation of reactive oxygen species and antioxidant defense in plants under salinity. Int. J. Mol. Sci..

[26] Zulfiqar B., Raza M.A.S., Saleem M.F., Ali B., Aslam M.U., Al-Ghamdi A.A., Elshikh M.S., Hassan M.U., Toleikienė M., Ahmed J. (2024). Abscisic acid improves drought resilience, growth, physio-biochemical and quality attributes in wheat (*Triticum aestivum* L.) at critical growth stages. Sci. Rep..

[27] Reddy K.R., Bheemanahalli R., Saha S., Singh K., Lokhande S.B., Gajanayake B., Read J.J., Jenkins J.N., Raska D.A., Santiago L.M.D. (2020). High-temperature and drought-resilience traits among interspecific chromosome substitution lines for genetic improvement of upland cotton. Plants.

[28] Galeano E., Vasconcelos T.S., Novais de Oliveira P., Carrer H. (2019). Physiological and molecular responses to drought stress in teak (*Tectona grandis* Lf). PLoS ONE.

[29] Vadez V., Grondin A., Chenu K., Henry A., Laplaze L., Millet E.J., Carminati A. (2024). Crop traits and production under drought. Nat. Rev. Earth Environ..

[30] Wang F., Li S., Kong F., Lin X., Lu S. (2023). Altered regulation of flowering expands growth ranges and maximizes yields in major crops. Front. Plant Sci..

[31] Kumar A., Sheoran P., Mann A., Yadav D., Kumar A., Devi S., Kumar N., Dhansu P., Sharma D.K. (2023). Deciphering trait associated morpho-physiological responses in pearlmillet hybrids and inbred lines under salt stress. Front. Plant Sci..

[32] Meshram J.H., Singh S.B., Raghavendra K.P., Waghmare V.N. (2022). Drought stress tolerance in cotton: Progress and perspectives. Climate Change and Crop Stress.

[33] Croce R., Carmo-Silva E., Cho Y.B., Ermakova M., Harbinson J., Lawson T., McCormick A.J., Niyogi K.K., Ort D.R., Patel-Tupper D. (2024). Perspectives on improving photosynthesis to increase crop yield. Plant Cell.

[34] Tang C.-j., Luo M.-Z., Zhang S., Jia G.-Q., Sha T., Jia Y.-C., Hui Z.H.I., Diao X.-M. (2023). Variations in chlorophyll content, stomatal conductance, and photosynthesis in Setaria EMS mutants. J. Integr. Agric..

[35] Simkin A.J., Kapoor L., Doss C.G.P., Hofmann T.A., Lawson T., Ramamoorthy S. (2022). The role of photosynthesis related pigments in light harvesting, photoprotection and enhancement of photosynthetic yield in planta. Photosynth. Res..

[36] Kaur H., Kohli S.K., Khanna K., Bhardwaj R. (2021). Scrutinizing the impact of water deficit in plants: Transcriptional regulation, signaling, photosynthetic efficacy, and management. Physiol. Plant..

[37] Al-Ashkar I., Alderfasi A., Ben Romdhane W., Seleiman M.F., El-Said R.A., Al-Doss A. (2020). Morphological and genetic diversity within salt tolerance detection in eighteen wheat genotypes. Plants.

[38] Miglani G.S., Kaur R., Sharma P., Gupta N. (2021). Leveraging photosynthetic efficiency toward improving crop yields. J. Crop Improv..

[39] Zhuang J., Wang Y., Chi Y., Zhou L., Chen J., Zhou W., Song J., Zhao N., Ding J. (2020). Drought stress strengthens the link between chlorophyll fluorescence parameters and photosynthetic traits. PeerJ.

[40] Motallebinia S., Sofalian O., Asghari A., Rasoulzadeh A., Achachlouei B.F. (2024). Genetic diversity and morpho-physiological assessment of drought tolerance in rapeseed (*Brassica napus* L.) cultivars. Plant Genet. Resour..

[41] Iqbal N., Rahman M.M., Cawthray G.R., Zhou Y., Denton M.D., Sadras V.O. (2024). Drought and Herbivory Differentially Modulate the Leaf Exudation of Organic Acids in Chickpea. J. Soil Sci. Plant Nutr..

[42] Choudhury S., Moulick D., Ghosh D., Soliman M., Alkhedaide A., Gaber A., Hossain A. (2022). Drought-induced oxidative stress in pearl millet (*Cenchrus americanus* L.) at seedling stage: Survival mechanisms through alteration of morphophysiological and antioxidants activity. Life.

[43] Zhang Y., Liu G., Dong H., Li C. (2021). Waterlogging stress in cotton: Damage, adaptability, alleviation strategies, and mechanisms. Crop J..

[44] Wang Y., Qi H., Liu Y., Duan C., Liu X., Xia T., Chen D., Piao H.-l., Liu H.-X. (2021). The double-edged roles of ROS in cancer prevention and therapy. Theranostics.

[45] Rajput V.D., Harish, Singh R.K., Verma K.K., Sharma L., Quiroz-Figueroa F.R., Meena M., Gour V.S., Minkina T., Sushkova S. (2021). Recent developments in enzymatic antioxidant defence mechanism in plants with special reference to abiotic stress. Biology.

[46] Hickey K., Wood M., Sexton T., Sahin Y., Nazarov T., Fisher J., Sanguinet K.A., Cousins A., Kirchhoff H., Smertenko A. (2022). Drought tolerance strategies and autophagy in resilient wheat genotypes. Cells.

[47] Omar A.A., Heikal Y.M., Zayed E.M., Shamseldin S.A.M., Salama Y.E., Amer K.E., Basuoni M.M., Abd Ellatif S., Mohamed A.H. (2023). Conferring of drought and heat stress tolerance in wheat (*Triticum aestivum* L.) genotypes and their response to selenium nanoparticles application. Nanomaterials.

[48] Nkonya E., Kato E. (2020). Rethinking agro-food sector to combat land degradation and desertification. Life on Land.

[49] Raza A., Mubarik M.S., Sharif R., Habib M., Jabeen W., Zhang C., Chen H., Chen Z.H., Siddique K.H.M., Zhuang W. (2023). Developing drought-smart, ready-to-grow future crops. Plant Genome.

[50] Magwanga R.O., Lu P., Kirungu J.N., Cai X., Zhou Z., Agong S.G., Wang K., Liu F. (2020). Identification of QTLs and candidate genes for physiological traits associated with drought tolerance in cotton. J. Cotton Res..

[51] Flores-Saavedra M., Villanueva G., Gramazio P., Vilanova S., Mauceri A., Abenavoli M.R., Sunseri F., Prohens J., Plazas M. (2024). Nitrogen use efficiency, growth and physiological parameters in different tomato genotypes under high and low N fertilisation conditions. Plant Physiol. Biochem..

[52] Gul N., Khan Z., Shani M.Y., Hafiza B.S., Saeed A., Khan A.I., Shakeel A., Rahimi M. (2025). Identification of salt-resilient cotton genotypes using integrated morpho-physiological and biochemical markers at the seedling stage. Sci. Rep..

[53] Hussain T., Akram Z., Shabbir G., Manaf A., Ahmed M. (2021). Identification of drought tolerant Chickpea genotypes through multi trait stability index. Saudi J. Biol. Sci..

[54] Pandey A., Khobra R., Mamrutha H.M., Wadhwa Z., Krishnappa G., Singh G., Singh G.P. (2022). Elucidating the drought responsiveness in wheat genotypes. Sustainability.

[55] Yahaya M.A., Shimelis H. (2022). Drought stress in sorghum: Mitigation strategies, breeding methods and technologies—A review. J. Agron. Crop Sci..

[56] Giannopolitis C.N., Ries S.K. (1977). Superoxide dismutases: I. Occurrence in higher plants. Plant Physiol..

[57] Chance B., Maehly A.C. (1955). [136] Assay of Catalases and Peroxidases.

[58] Erel O. (2004). A novel automated method to measure total antioxidant response against potent free radical reactions. Clin. Biochem..

[59] Cakmak I., Horst W.J. (1991). Effect of aluminium on lipid peroxidation, superoxide dismutase, catalase, and peroxidase activities in root tips of soybean (*Glycine max*). Physiol. Plant..

[60] Velikova V., Yordanov I., Edreva A. (2000). Oxidative stress and some antioxidant systems in acid rain-treated bean plants: Protective role of exogenous polyamines. Plant Sci..

[61] Bradford M.M. (1976). A rapid and sensitive method for the quantitation of microgram quantities of protein utilizing the principle of protein-dye binding. Anal. Biochem..

[62] Riazi A., Matsuda K., Arslan A. (1985). Water-stress induced changes in concentrations of proline and other solutes in growing regions of young barley leaves. J. Exp. Bot..

[63] Bates L.S., Waldren R., Teare I.D. (1973). Rapid determination of free proline for water-stress studies. Plant Soil.

[64] Arnon D.I. (1949). Copper enzymes in isolated chloroplasts. Polyphenoloxidase in Beta vulgaris. Plant Physiol..

[65] Irnawati I., Riswanto F.D.O., Riyanto S., Martono S., Rohman A. (2021). The use of software packages of R factoextra and FactoMineR and their application in principal component analysis for authentication of oils. Indones. J. Chemom. Pharm. Anal..

[66] Gu Z., Hübschmann D. (2022). Make interactive complex heatmaps in R. Bioinformatics.

[67] Olivoto T., Nardino M. (2021). MGIDI: Toward an effective multivariate selection in biological experiments. Bioinformatics.

